# Childhood Maltreatment Influences Autonomic Regulation and Mental Health in College Students

**DOI:** 10.3389/fpsyt.2022.841749

**Published:** 2022-06-02

**Authors:** Lourdes P. Dale, Jacek Kolacz, Jennifer Mazmanyan, Kalie G. Leon, Karli Johonnot, Nadia Bossemeyer Biernacki, Stephen W. Porges

**Affiliations:** ^1^Department of Psychiatry, College of Medicine-Jacksonville, University of Florida, Jacksonville, FL, United States; ^2^Traumatic Stress Research Consortium, Kinsey Institute, Indiana University, Bloomington, IN, United States; ^3^Socioneural Physiology Laboratory, Kinsey Institute, Indiana University, Bloomington, IN, United States; ^4^Department of Psychology, University of Hartford, West Hartford, CT, United States; ^5^Department of Psychology, University of North Florida, Jacksonville, FL, United States; ^6^Department of Psychiatry, School of Medicine, University of North Carolina at Chapel Hill, Chapel Hill, NC, United States

**Keywords:** maltreatment, PTSD, heart rate variability, respiratory sinus arrhythmia, Polyvagal Theory, vagal efficiency

## Abstract

Childhood maltreatment history may influence autonomic reactivity and recovery to stressors. Hypothetically, the maltreatment history may contribute to a retuned autonomic nervous system that is reflected in a novel metric, vagal efficiency (VE), designed to assess the functional efficiency of vagal cardioinhibitory pathways on heart rate. We explored whether VE mediates the well-documented relationship between maltreatment history and psychiatric symptoms. We also investigated the relationship between measures of autonomic regulation in response to the physical and emotional challenges and psychiatric symptoms. Participants (*n* = 167) completed self-report measures of psychiatric symptoms and had continuous beat-to-beat heart rate monitored before, during, and after physical and emotional stressors. Participants with maltreatment histories exhibited lower VE, which mediated the association of maltreatment history and the psychiatric symptoms of anxiety and depression. Consistent with prior literature, there were significant associations between maltreatment history and autonomic reactivity (i.e., heart rate and respiratory sinus arrhythmia) during emotional and physical challenges; however, when VE was entered as a covariate these associations were no longer statistically significant. Blunted VE may reflect a neural pathway through which maltreatment retunes autonomic regulation and provides a neurophysiological platform that increases mental health risk.

## Introduction

Exposure to traumatic events may have psychological and physiological consequences that may be a result of dysregulation of the autonomic nervous system. Survivors of maltreatment, even those who do not reach the diagnostic criteria for PTSD, may have psychiatric and physical health features that relate to an autonomic nervous system that has been retuned to have a lower threshold to react to cues of threat (1–4). Prior research has shown that female college students with maltreatment histories, who did not reach the diagnostic criterion for PTSD, experienced more psychiatric symptoms, had lower levels of respiratory sinus arrythmia (RSA) and faster heart rates as demonstrated by their shorter heart periods (HP), and reacted differently to the physical stressor of riding a stationary bike and the emotional stressor of watching a video of a child being maltreated than women without maltreatment histories (5, 6). Additionally, meta-analyses found major depression and anxiety disorders such as panic disorder, PTSD, generalized anxiety disorder, and social anxiety are associated with lower heart rate variability (HRV) (7, 8). These patterns of autonomic reactivity and recovery to stressors might relate to atypical vagal regulation of the heart reflected in inefficient cardiohibitory vagal pathways (i.e., vagal brake) (6).

Polyvagal Theory (9, 10) proposes that these protective responses spontaneously emerge through neuroception, a reflexive adaptive process that triggers specific biobehavioral response patterns to cope with conditions of safety, danger, and life threat. If neuroception detects safety, the nervous system facilitates social communication and engagement. In contrast, if neuroception detects danger, the withdrawal of the parasympathetic system may be initiated, reducing the impact of cardioinhibitory vagal pathways on the heart to functionally diminish the influence of the vagal brake on the heart’s pacemaker and increase access to greater metabolic resources in preparation for the challenge. Following a successful response to the challenge, the parasympathetic nervous system initiates recovery by re-engaging the vagal brake to slow heart rate by increasing the cardioinhibitory actions of the vagus, while simultaneously inhibiting the sympathetic control of the heart (11, 12). Since the early 1900s, it had been known that the cardioinhibitory function of the vagus was systematically influenced by respiration (13). This observation is the physiological basis for quantifying the amplitude of the oscillation in heart rate at the frequency of spontaneous breathing, as an index of cardiac vagal tone (14). Lower levels of RSA have been associated with a greater sensitivity to unpredictable threat (15), a finding that relates to neuroception and has implications for individuals with a history of maltreatment.

In this study, we explored this plausible explanation using vagal efficiency (VE), a metric proposed by Porges and colleagues (16) as a measure of the dynamic regulation of cardiac vagal tone on cardiac output represented in a single measure of slope between sequential measures of HP and RSA. We examined VE during the physical challenge of riding a stationary bike because it provided a physiological challenge with minimal potential psychological associations. In addition, the metabolic demands of biking require a systematic withdrawal of the vagal cardioinhibitory influence on the heart’s pacemaker (i.e., vagal brake), while the post-biking recovery enables the vagal brake to re-engage to slow heart rate. If the heart rate is tightly coupled and efficiently driven by the vagal brake (i.e., measured by RSA), the linear regression between short (e.g., 15s) sequential estimates of RSA and heart rate will have a steep slope.

Although no published studies have examined how VE relates to maltreatment history and psychiatric symptoms, it is important to consider VE as it may be developmentally sensitive to environmental conditions. In preterm infants, VE has been found to increase with maturation and to be influenced by a psychosocial intervention (17). Furthermore, its sensitivity to environmental conditions was demonstrated as VE was reliably reduced following the administration of alcohol in adults (18). VE may also predict intervention response, with evidence showing that adolescents with low VE were more responsive to neurostimulation for their gastric pain than those whose VE was high (19).

Consistent with Polyvagal Theory (9–11, 20, 21), which emphasizes the mediational role of autonomic state as an intervening variable, it is important to investigate autonomic reactions to an emotional stressor because exposure to traumatic events may retune autonomic regulation and contribute to dysfunctional emotional processing (22, 23). These individuals may adaptably become hypervigilant for danger cues and have anticipatory or responsive physiological changes in their autonomic nervous system that promote defensive action and may influence psychological wellbeing. When confronted with emotional challenges, including emotional imagery and emotional scene viewing, individuals exposed to stressful events have consistently showed atypical physiological and neural responses (24–29). We also investigated autonomic responses during the emotional stressor of watching a video of a child being maltreated and contrasted it to the reaction during the physical stressor.

The current study explored whether VE was reduced in those with a maltreatment history, and whether it mediated the relationship between maltreatment history and psychiatric symptoms (i.e., somatization, anxiety, depression, and PTSD). We also investigated whether VE mediated the relationship between measures of autonomic regulation in response to the physical and emotional challenges and psychiatric symptoms. We hypothesized that:

• Participants with a history of maltreatment would have lower VE and exhibit autonomic regulation difficulties in response to the physical and emotional stressor challenges.
• Measures of VE and autonomic reactivity and recovery would be correlated with psychiatric symptoms.
• VE would mediate the relationship between autonomic reactivity and psychiatric symptoms.

## Materials and Methods

### Participants

Participants were 167 college students (65.9% identified as female), recruited from a university’s participant pool for two separate studies, who had data for all the physiological measures. They were 18–25 years old (*M* = 19.18, *SD* = 1.33), predominantly first-year (45.5%) or second year (25.7%) students. The racially diverse sample identified themselves as White (43.7%), Black (16.8%), Hispanic (9.0%), Asian/Pacific Islander (6.0%), and mixed/other (24.6%). Those reporting a medical diagnosis (8.4%) did not report having a cardiovascular disorder, which would have excluded them from participation in this study.

Participants with a prior psychiatric diagnosis (25.1%) most frequently reported having anxiety (15.6%), depression (14.4%), ADHD (8.4%), and PTSD (2.4%). There were no participants that did not endorse at least mild maltreatment for at least one of the items. Many reported experiencing moderate or severe childhood maltreatment (48.5%) including emotional neglect (31.1%), emotional abuse (28.1%), physical neglect (18.6%), physical abuse (16.8%), and sexual abuse (13.2%). Participants varied in their current symptomatology (non-windsorized values for somatization *M* = 3.27, *SD* = 4.01; depression *M* = 4.57, *SD* = 5.07; anxiety *M* = 4.93, *SD* = 5.14; and PTSD *M* = 30.33, *SD* = 10.49) and those reporting more of one symptom reported more of another (correlations ranged 0.52–0.75, *p* < 0.001).

### Procedure

The university’s Institutional Review Board approved all procedures. During the data collection sessions, the participant was provided with an informed consent form explaining the voluntary nature and purpose of the study, participation requirements, and confidentiality and privacy procedures. Each participant was informed there were no known physical or psychological risks from participating in this study and that the investigator should be notified if they were distressed. Once written consent was obtained, the participant completed self-report measures with the least sensitive/personal information being asked first and the most sensitive/personal information last. Then the participant was asked their height and weight and instructed on how to attach the electrodes for the heart rate monitoring. All participants were first exposed to the physical stressor of riding the stationary bike at a comfortable pace for half a mile (usually 2–4 min) and then the emotional stressor of watching a video in which a child is emotionally maltreated (3 min). Data were collected during a 3-min baseline prior to and after each stressor to enable the quantification of changes in heart rate and respiratory sinus arrhythmia to the experimental challenges.

As a precaution, the instruments assessing symptoms of depression and PTSD were scored immediately to determine if the participant reported having in the past 7 days any desire to end their life and being extremely hopeless about the future and endorsed a clinically significant level of traumatic stress (scored 44 or higher on the measure of PTSD symptomatology). This protocol identified about 30% of the participants that needed an immediate assessment to determine level of risk by the licensed clinical psychologist that was part of the research team. All participants were referred to the counseling center and provided with an information sheet that explained other relevant university services (e.g., academic support services). Because none of these participants required psychiatric hospitalization, they all remained in the study. After data collection, participants were provided with a debriefing form that had information about the study and resources on campus.

### Constructs and Measures

**Maltreatment history** was assessed via the Childhood Trauma Questionnaire (30), which is a 28-item self-report questionnaire that asks adolescents and adults about how often they experienced emotional abuse, sexual abuse, physical abuse, emotional neglect, and physical neglect. This measure is internally consistent (α = 0.66–0.93), reliable (test-retest *r* = 0.86), and converges with corroborated clinician reports of maltreatment history (31, 32). A binary childhood maltreatment score was derived by determining if the individual reported moderate or severe maltreatment in any of the five domains.

**Psychiatric symptoms** were assessed via two measures. Depression, anxiety, and somatization symptoms were assessed via the 18-item version of the Brief Symptom Inventory (33), which asks participants to indicate via a 5-point Likert scale (*not at all* to *extremely*) how much they have been distressed or bothered in the past 7 days by each symptom. Reliability analyses indicated that the measure was internally consistent with the current sample (Cronbach’s alpha scores somatization = 0.81; anxiety = 0.87, and depression = 0.88).

PTSD symptoms were assessed via the PTSD Checklist—Civilian Version (34), a 17-item self-report measure that corresponds to the criteria for PTSD and assesses via a 5-point Likert scale (*not at all* to *extremely*) level of distress related to stressful life experiences over the last month. This measure has good convergent and discriminant validity, internal consistency, and test–retest reliability (35) and was found to be internally consistent with the current sample (Cronbach’s alpha = 0.92).

**Physiological data** were monitored with an EZ-IBI monitor (UFI, Morro Bay, CA). Two active electrodes were placed on the left side at the level of the heart and on the right lower abdomen. A ground electrode was placed above the right-side collarbone. ECG data was collected continuously while the participant went through the protocol. The EZ-IBI detected the peak of the R-wave with 1-ms accuracy (sampling rate = 1,000 Hz) and timed the interval ms between sequential R-R waves (i.e., heart period, HP), which were downloaded to a computer for off-line processing and analyses. Files of sequential HP were stored on a computer. HP was used as the metric of heart rate (i.e., HP is monotonically related to heart rate).

Quantification of heart rate variability requires the timing of intervals between successive heart beats synchronized with successive peaks of the R-wave in the ECG. In this paper, we label the R-R intervals defining interbeat intervals, as heart period. We elected to report the data using heart period instead of heart rate because heart period reflects a stronger linear relationship than heart rate with dynamic vagal influences (36).

RSA estimates calculated based on the methods developed by Porges (37) included the following procedures: (a) the R-R interval time series were converted to time-based data by resampling at successive 500-ms intervals; (b) a 21-point moving cubic polynomial filter was stepped through the time-sampled series to produce a smoothed template series; (c) the template series was subtracted from the original series to produce a residual time series; (d) the residual time series was processed by a digital bandpass filter with 25 coefficients to extract the variance in the frequency band of 0.12–0.40 Hz (i.e., frequency of spontaneous breathing for adults); and (e) bandpassed variance was transformed to its natural logarithm and used to quantify RSA. These procedures result in a sensitive, non-invasive marker of the influence of the myelinated vagal fibers on the heart (9, 11, 13, 38).

Data files were input into CardioEdit software (39) to visually display sequential R-R interval data and edit outliers. Edited data were processed with CardioBatch software (39) to generate measures of mean 30-s epochs for HP and RSA.

Baseline levels were based on the first segment collected. Autonomic metrics reflected the magnitude of change in RSA and HP from baseline to stressor (reactivity) and stressor to post-stressor (recovery) for the physical and emotional stressors. Reactivity change scores were calculated by subtracting the value during the pre-stressor baseline from the value during the stressor. Recovery change scores were calculated by subtracting the value during the stressor from the value during the post-stressor recovery period.

VE was used to measure cardiac autonomic regulation, specifically how vagal efferent pathways to the heart dynamically influences heart rate, which is a process that is not captured in RSA alone (19). Following methods from prior studies (19, 40), VE was calculated for each participant as the slope of the regression line between RSA and HP using paired sequential epoch values (every 15 s) of HP and RSA during the physical challenge (i.e., 3-min baseline, riding the stationary bike, and post-biking recovery).

### Statistical Analysis

Data analyses were conducted in SSPS and R. To retain participants and minimize the effects of symptomatology outliers, a 90% windsorization procedure was used. Correlational analyses explored the relationship among the autonomic measures (i.e., VE, baseline levels and changes in RSA and HP in response to physical and emotional stressors) and psychiatric symptomatology. Independent sample *t*-tests compared participant groups with and without self-reported history of maltreatment.

Mediation analyses, conducted using the Lavaan R package (41), assessed whether the association between the independent variable of maltreatment history (yes/no) and dependent variables of psychiatric symptoms could be attributed to the indirect effect of the third variable of VE. For this analysis, the indirect effect, which represents the strength of the mediation, is the product of the coefficient of the independent variable on the mediator and the mediator on the outcome variable and the direct effect is the effect of the independent variable on the dependent variable adjusting for the effect of the mediator. Mediation models were estimated using maximum likelihood. Indirect and total effect confidence intervals were calculated using bias-corrected adjusted bootstrap percentiles with 10,000 draws, which has superior power for detecting true effects with accurate Type I error rates compared to other methods (42). Mediation was supported if the indirect effect 95% confidence interval did not include zero.

The final analyses did not focus on maltreatment history. Instead, the repeated measures analyses explored whether participants who scored above and below the clinical cutoff scores for somatization, depression, anxiety, and PTSD symptoms varied in their HP changes during the physical and emotional stressor challenges. We also explored whether these effects remained when VE was entered as a covariate.

## Results

Distributions of the autonomic variables (i.e., VE, HP, and RSA) were explored for outliers. One extreme outlier on the VE measure (VE = 280.99, 6 *SD* above mean) was removed from the analysis. With this exclusion, VE scores ranged from 22.37 to 158.61 (*M* = 66.06, *SD* = 23.38). Table 1 provides the descriptive statistics for the baseline and changes scores [natural log (ln) units for RSA and ms for HP].

**TABLE 1:** Descriptive statistics for RSA and HP.

	RSA			HP		
Variable	*M*	Min	Max	*M*	Min	Max
Baseline	7.18 (1.14)	3.55	9.78	861.60 (143.52)	544.00	1320.68
**Physical stressor**						
Reactivity	−4.52 (1.63)	−8.78	0.02	−370.17 (130.09)	−873.67	−82.43
Recovery	3.48 (1.43)	0.40	6.99	222.55 (105.24)	47.46	561.59
**Emotional stressor**						
Reactivity	−0.02 (0.90)	−4.95	3.52	40.32 (60.06)	−271.83	245.03
Recovery	0.09 (0.63)	−1.55	3.11	−9.78 (58.54)	−180.16	480.88

### Vagal Efficiency

**FIGURE 1:**
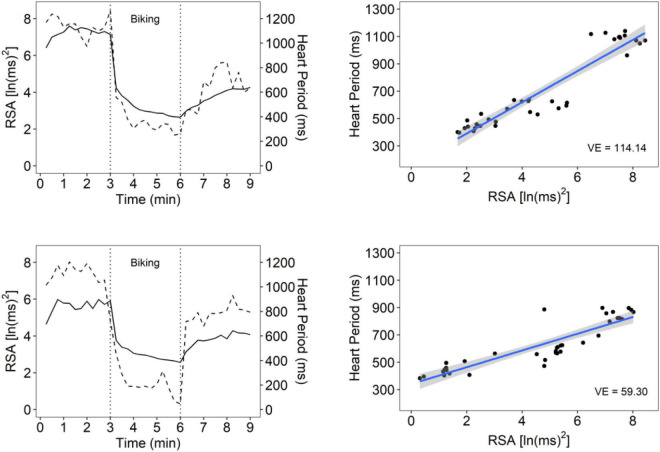
An illustration of vagal efficiency (VE) during baseline, biking, and recovery. Data is displayed for 2 participants: one with high VE (top row) and one with low VE (bottom row). Figures in the left column show cardiac vagal tone [measured by respiratory sinus arrhythmia (RSA); dotted line] and HP (solid line) over the course of the protocol. The participant in the top row has strong coupling between RSA and HP, which can also be seen in the steep slope between synchronous observations in the top right figure (slope = 114.14). The top right plot reflects the participants’ rapid shift from high RSA/high HP during baseline and recovery relative to low RSA/low HP states during physical exercise. These data suggest a rapid withdrawal and re-engagement of the vagal brake to regulate metabolic demands. The participant in the bottom panel had similar ranges of RSA values, but RSA was weakly associated with HP (slope = 59.30), suggesting that cardioinhibitory vagal influences were not efficient in slowing cardiac metabolic output.

Figure 1 illustrates VE for a participant with high VE and one with low VE. Table 2 documents that participants with a maltreatment history exhibited dampened autonomic regulation reflected in lower RSA and HP baseline measures and lower VE. In addition, the participants with a maltreatment history reported more psychiatric symptoms.

**TABLE 2:** Comparison of maltreatment and no maltreatment groups.

	Maltreatment group			No maltreatment group			*T_1,165_*	*P*	*D*
	*M* (*SD*)	95% CI		*M* (*SD*)	95% CI				
		Lower	Upper		Lower	Upper			
Vagal efficiency	61.70 (19.48)	57.40	66.01	69.56 (25.54)	0.35	75.70	−**2.22**	**0.028**	0.37
Baseline RSA	6.97 (1.23)	6.69	7.24	7.38 (1.02)	0.37	7.60	−**2.35**	**0.020**	0.38
Baseline HP	831.70 (136.80)	801.45	861.95	888.79 (145.35)	0.40	920.14	−**2.60**	**0.010**	0.42
**Physical stressor**									
RSA reactivity	−4.71 (1.53)	−0.5.04	−4.37	−4.38 (1.68)	0.21	−4.01	−1.32	0.190	0.21
HP reactivity	−357.62 (129.28)	−386.20	−329.03	−382.68 (131.17)	−0.19	−354.22	1.24	0.218	−0.22
RSA recovery	3.47 (1.35)	3.18	3.77	3.51 (1.49)	0.03	3.84	−0.18	0.859	0.01
HP recovery	198.96 (96.70)	177.58	220.34	244.11 (108.96)	0.44	267.76	−**2.81**	**0.006**	0.45
**Emotional stressor**									
RSA reactivity	0.11 (0.90)	−0.09	0.31	−0.13 (0.90)	−0.27	0.06	1.72	0.087	−0.41
HP reactivity	32.78 (64.79)	18.45	47.11	49.06 (52.98)	0.28	60.56	−1.77	0.079	0.25
RSA recovery	0.01 (0.53)	−0.11	0.14	0.16 (0.70)	0.24	0.32	−1.50	0.136	0.19
HP recovery	−5.45 (40.00)	−14.41	3.51	−14.67 (71.80)	−0.29	1.01	1.00	0.318	−0.29
**Symptoms**									
Somatization	3.85 (4.13)	2.94	4.76	2.37 (2.62)	0.43	2.94	**2.75**	**0.007**	−0.43
Depression	5.77 (5.10)	4.64	6.89	3.16 (3.90)	0.57	4.02	**3.65**	**0.000**	−0.57
Anxiety	5.94 (4.97)	4.84	7.04	3.59 (4.05)	0.52	4.46	**3.30**	**0.001**	−0.53
PTSD	34.78 (13.28)	31.82	37.73	27.22 (9.40)	0.66	29.25	**4.20**	**0.000**	−0.53

*D = Cohen’s d. Bold values indicate significant group differences (p < 0.05).*

**FIGURE 2:**
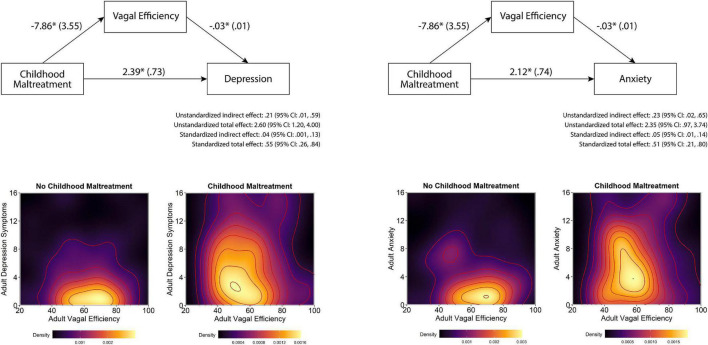
Effects of childhood maltreatment on depression and anxiety as mediated by vagal efficiency. Note that in the top figures, first numbers represent path estimates and parentheses reflect standard errors, with significant paths marked with *. Partial mediation was supported by a non-zero indirect effects. In the bottom figures are density plots of VE and depression and anxiety symptoms, with lighter colors indicating higher density of observations and darker colors representing fewer observations. The modal tendency of the maltreatment sample skews left indicating poorer VE and upward indicating higher depression and anxiety symptomatology compared to the non-maltreated sample on the left.

Independent of maltreatment history, participants with lower VE reported more symptoms of depression and anxiety (Table 3). As illustrated in Figure 2, mediation analyses assessing whether the association between maltreatment history and the symptoms of depression and anxiety could be attributed to VE found evidence of partial mediation for both depression and anxiety. The indirect effect in both models was significant, with childhood maltreatment history being associated with lower VE, which—in turn—was associated with higher levels of anxiety and depression symptoms (Figure 2 top panels). Plots of raw distributions showed that participants with childhood maltreatment had higher scores in adult depressive symptoms and adult anxiety that were associated with lower VE. This was in contrast to participants without childhood maltreatment history who had higher VE and concurrently lower levels of anxiety and depression symptoms (Figure 2 bottom panels).

**TABLE 3:** Correlation among physiological measures and symptomatology.

	Somatization symptoms	Depression symptoms	Anxiety symptoms	PTSD symptoms
Vagal efficiency	–0.05	−0.16*	−0.17*	–0.10
**Baseline**				
Baseline RSA	0.01	0.05	–0.05	0.09
Baseline HP	–0.09	–0.15	−0.20**	–0.12
**Physical stressor**				
RSA reactivity	–0.02	–0.05	–0.03	–0.05
HP Reactivity	0.07	0.13	0.13	0.12
RSA recovery	–0.01	0.00	0.02	0.10
HP recovery	−0.18*	−0.17*	−0.21**	–0.11
**Emotional stressor**				
RSA reactivity	0.07	0.09	0.09	0.10
HP reactivity	−0.16*	−0.19**	−0.24**	−0.17*
RSA recovery	0.02	–0.11	–0.09	–0.08
HP recovery	0.01	0.05	0.03	–0.03

*p < 0.05, **p < 0.01.

### Maltreatment History, ANS Regulation, and Symptomatology

As reported in Table 4, VE was correlated with baseline levels, and HP reactivity and recovery to the physical stressor. The negative correlation between VE and HP reactivity indicates that participants with higher VE exhibited a greater reduction in HP in response to the physical stressor, which suggests greater increases in heart rate. Whereas, the positive correlation between VE and HP recovery indicates that participants with higher VE exhibited a greater increase in HP after the physical stressor, which suggests greater slowing of their heart rates.

**TABLE 4:** TableCorrelations among physiological variables.

	Physical stressor				Emotional stressor			
	Reactivity		Recovery		Reactivity		Recovery	
	RSA	HP	RSA	HP	RSA	HP	RSA	HP
Vagal efficiency	0.15	−0.51***	–0.10	0.48***	–0.03	0.07	0.04	0.04
**Physical stressor**								
RSA reactivity		0.45***	−0.45***	0.15	−0.26**	–0.14	0.16*	–0.03
HP reactivity			−0.35***	−0.45***	0.06	−0.21**	−0.16*	0.05
RSA recovery				0.48***	–0.09	0.04	−0.16*	–0.09
HP recovery					−0.34***	0.00	0.06	–0.14
**Emotional stressor**								
RSA reactivity						0.42***	−0.46***	–0.10
HP reactivity							–0.08	−0.41***
RSA recovery								0.40***

*Vagal efficiency was calculated for the physical stressor.*

*p < 0.05, **p < 0.01, ***p < 0.001.

**FIGURE 3:**
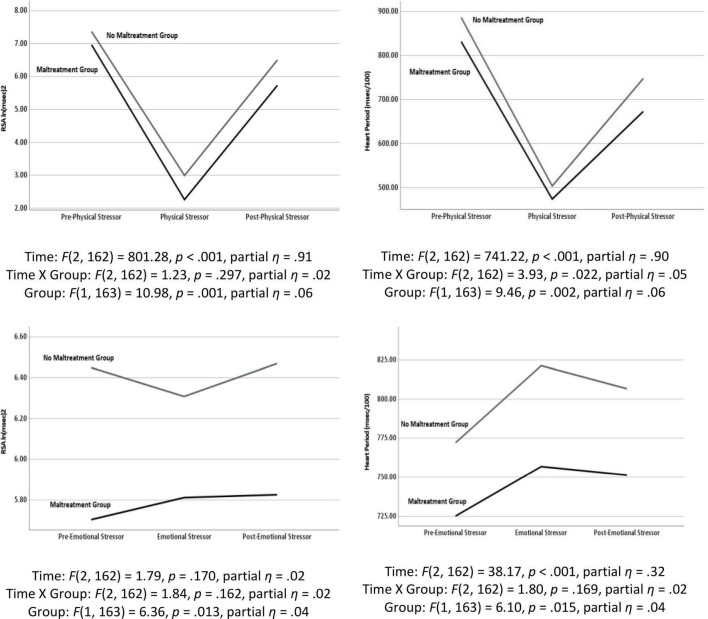
Results of repeated measures analyses of variance documenting changes in respiratory sinus arrhythmia (RSA) and heart period (HP) in response to the physical stressor (i.e., riding a stationary bike) and the emotional stressor (i.e., viewing a video of a child being maltreated) as a function of maltreatment history.

As illustrated in Figure 3, the repeated measures were significantly different during the physical challenge for RSA and HP. During the emotional challenge, only HP systematically changed during the protocol. Note in the figure the significant between group differences with the maltreatment group having consistently lower levels of RSA and HP. In addition, a significant maltreatment group X repeated measures interaction documented that the HP reaction during the physical challenge significantly differed between the groups. As reported in Table 2, the participants with histories of maltreatment exhibited significantly less HP recovery in response to the physical stressor and less RSA reactivity in response to the emotional stressor.

As reported in Table 3, only measures of HP reactivity and recovery were associated with psychiatric symptoms. Participants with lower baseline HP levels reported more anxiety and PTSD symptoms; less HP recovery from the physical stressor reported more somatization, depression, and anxiety symptoms; and less HP reactivity in response to the emotional stressor reported more somatization, depression, anxiety, and PTSD symptoms. However, consistent with the mediation analyses, when VE was included as a covariate most correlations were no longer significant. The only remaining significant relationships were between HP recovery to the physical stressor and anxiety symptoms (*r* = −0.25, *p* = 0.027) and HP reactivity to the physical stressor and anxiety and PTSD symptoms (*r* = 0.23, *p* = 0.042 and *r* = 0.35, *p* = 0.002).

### Exploring Pathways of Mediation

Table 5 displays the results of repeated measures analyses exploring whether participants who scored above and below the clinical cutoff scores for somatization, depression, anxiety, and PTSD symptoms varied in their HP changes during the physical and emotional stressor challenges. Specifically, we looked for main effects related to group status and interaction effects that considered the interaction of group status and HP levels. We also explored whether these effects remained when VE was entered as a covariate. As evident in Table 5, participants scoring above and below the clinical cutoffs for psychiatric symptomatology differed in the HP response to the physical and emotional stressor challenges. When VE was used as a covariate in analyses evaluating the autonomic responses to the physical stressor challenge for groups defined as above or below the clinical cutoffs for somatization, depression, anxiety, and PTSD the observed clinically related shorter HP was no longer statistically significant. In addition, for the groups defined by being below and above the clinical cutoffs for somatization, depression, anxiety, and PTSD there were significant group by repeated measures interactions. When VE was included as a covariate, the only significant interaction that remained was between the somatization groups. Similarly, with the emotional stressor challenge, the significant group differences related to depression, anxiety, and PTSD symptomatology were no longer present when VE was entered as a covariate. However, entering of VE as a covariate did not impact the HP by group interaction effects for the depression, anxiety, and PTSD groups (Table 5).

**TABLE 5:** Repeated measures ANOVA comparison of heart period regulation for the psychiatric symptom clinical cutoff groups.

Psychiatric symptom groups	Main effects for psychiatric groups and group × HP interaction effects	Without covariate			VE as Covariate		
		*F*	*p*	η^2^	*F*	*p*	η^2^
**HP during physical stressor challenge**							
Somatization	Group main effect	4.35	0.039	0.026	3.57	0.061	0.021
	Interaction effect	3.78	025	0.044	3.34	0.038	0.039
Depression	Group main effect	4.52	0.035	0.027	2.15	0.144	0.013
	Interaction effect	3.90	0.022	0.045	2.68	0.072	0.032
Anxiety	Group main effect	9.21	0.003	0.053	2.49	0.116	0.015
	Interaction effect	4.67	0.011	0.054	1.58	0.208	0.019
PTSD	Group main effect	5.06	0.026	0.030	1.96	0.163	0.012
	Interaction	3.40	0.036	0.040	1.83	0.165	0.022
**HP during emotional stressor challenge**							
Somatization	Group main effect	3.17	0.077	0.019	2.18	0.142	0.013
	Interaction effect	1.74	0.178	0.021	1.62	0.201	0.020
Depression	Group main effect	4.24	0.041	0.026	2.29	0.133	0.014
	Interaction effect	3.73	0.028	0.044	3.41	0.036	0.041
Anxiety	Group main effect	7.46	0.007	0.044	1.64	0.203	0.010
	Interaction effect	4.95	0.008	0.058	4.48	0.013	0.053
PTSD	Group main effect	5.31	0.022	0.032	2.66	0.105	0.016
	Interaction effect	3.81	0.024	0.046	3.53	0.032	0.043

## Discussion

In this study, we expanded prior research (6) that documented that maltreatment history was associated with inefficient or atypical vagal regulation of the heart in response to physical and emotional stressors. In this study, we investigated whether these findings relate to differences in VE. Consistent with our hypothesis, we documented that a history of maltreatment was related to lower levels of VE and that VE was related to dampened heart rate reactivity and recovery to the physical stressor.

Further support comes from the finding that participants with lower VE levels reported more depression and anxiety symptoms, and that VE mediated the relationship between maltreatment history and depression and anxiety symptoms. These findings are consistent with Polyvagal Theory (9–11, 20, 21), which proposes that autonomic state functions as an intervening variable mediating reactivity to challenges. The findings are also consistent with prior research (43) that documented that a subjective measure of autonomic reactivity (i.e., Body Perception Questionnaire) (44, 45) mediated the relationship between maltreatment history and current worry, depression, and PTSD symptoms during the pandemic. Future research will need to address whether subjective experiences of autonomic symptoms positively correspond with sensor-based measures such as VE.

Psychiatric symptoms were also related to metrics of autonomic regulation. Participants who exhibited less heart rate recovery in response to the physical stressor reported more somatization, depression, and anxiety symptoms, and those who exhibited less heart rate increases in response to the emotional stressor reported more somatization, depression, anxiety, and PTSD symptoms. Our findings provide a neurophysiological substrate for clinical observations that stress related disorders, such as anxiety, depression, and PTSD (46), are often marked by heightened reactivity and difficulty self-regulating while attempting to adaptively function (47) and adjust to environmental circumstances.

The results are consistent with prior research suggesting that major depression, anxiety disorders, and PTSD are associated with lower HRV (7, 8). Furthermore, the findings of an association between psychiatric symptoms and autonomic regulation builds on prior research that found those with lower vagal tone maintained consistently low HRV during a stress task and exhibited no post-stress recovery (48). Additionally, the results support previous research suggesting those with psychiatric disorders have less vagal activation and distinct, dysregulated autonomic profiles compared to healthy controls as demonstrated through measures of HRV and RSA (49). Thus, measures of heart rate regulation, particularly recovery from a physical stressor and reactivity to an emotional stressor, may be useful in assessing difficulties associated with the psychiatric components of PTSD. They may also be useful in measuring the autonomic dysregulation that may precede cardiovascular, autoimmune, or stress-related disease (48).

Repeated measures analyses indicated participants above the clinical cutoff for the psychiatric symptoms exhibited faster heart rates (i.e., shorter HP). Changes related to heart rate reactivity and recovery were greater in the group below the clinical cutoff for PTSD, suggesting that the enhanced range of reactivity reflected more efficient neural control of heart rate through vagal mechanisms. Because many group-related differences in autonomic regulation were removed when VE was entered as a covariate, VE may influence accessibility to efficiently regulate metabolic resources to rapidly adjust to transitory demands and impact on psychiatric health by mediating the individual’s ability to calm and socially engage.

Our findings are consistent with Polyvagal Theory (9–11), which proposes that following traumatic experiences the neural regulation of the “vagal brake” may become dysregulated (i.e., dampened) which may lead to less effective autonomic regulation and difficulties returning the body to a calm (i.e., ventral vagal regulated) baseline after experiencing a stressor. Several studies have reported an association between trauma history and atypical or disrupted autonomic functioning that leads to a heightened or potentially destabilized autonomic nervous system reflecting an inability to return to a more homeostatic state (50–52).

It is important to acknowledge that maltreatment history may also influence the sympathetic branch of the autonomic nervous system. Although no independent measure of sympathetic tone was monitored, a sympathetic contribution can be inferred from the heart rate data because neurophysiologically heart rate is determined by both sympathetic and vagal influences. If we assume that VE is effectively capturing the dynamic influence of vagal pathways on heart rate, then the use of VE as a covariate suggests that some of the remaining heart rate changes may be due to sympathetic influence. The results of covariate analyses support this speculation and document a relationship between clinical symptoms (anxiety and PTSD) and both HP reactivity and recovery to the physical challenge.

When interpreting these results, it is important to consider the limitations of this study. The use of a non-clinical sample of college students drawn from an introductory psychology participant pool at a private university necessitates the need for replication studies in non-college and clinical samples. In addition, maltreatment history and symptom data were obtained via self-report measures that may have been affected by social desirability and recall inaccuracies. Although the results are consistent with Polyvagal Theory, the cross-sectional design of this study does not allow us to determine whether VE is a causal determinant in the relationship between maltreatment history and symptomatology. Moreover, the lack of an independent measure of sympathetic activation limits interpretation of the dynamics between sympathetic and vagal influences. Thus, future research should be longitudinal, use larger and more diverse samples, conduct clinical interviews, obtain an independent measure of sympathetic tone, and use objective measures of symptomology.

Despite these limitations, the results highlight the importance of utilizing a biopsychosocial perspective when examining or predicting resilience, as both physiological and environmental factors relate to one’s functioning and experience of symptoms (53, 54), and may impact or alter one’s reactions to stress. The differences in autonomic regulation observed by those who experienced maltreatment suggest autonomic state regulation may be an important intervention target for trauma and PTSD, which could be supported by the inclusion of body-based, bottom-up methods as part of therapy (55, 56).

## Data Availability Statement

The original contributions presented in the study are included in the article/supplementary material, further inquiries can be directed to the corresponding author/s.

## Ethics Statement

The studies involving human participants were reviewed and approved by the University of Hartford. The patients/participants provided their written informed consent to participate in this study.

## Author Contributions

LD: oversee study planning, data collection and analyses, and write-up of manuscript. JK: data analyses, making figures, writing and editing of manuscript. JM: oversee data collection and data analyses. KL: editing of manuscript, fixing the references, and revising the manuscript after submission. KJ: writing of sections of manuscript. NB: creating of figures, editing of manuscript, and fixing of references. SP: conceptualization of manuscript, data analyses, and editing of manuscript. All authors contributed to the article and approved the submitted version.

## Conflict of Interest

The authors declare that the research was conducted in the absence of any commercial or financial relationships that could be construed as a potential conflict of interest.

## Publisher’s Note

All claims expressed in this article are solely those of the authors and do not necessarily represent those of their affiliated organizations, or those of the publisher, the editors and the reviewers. Any product that may be evaluated in this article, or claim that may be made by its manufacturer, is not guaranteed or endorsed by the publisher.
